# Copper Supplementation, A Challenge in Cattle

**DOI:** 10.3390/ani10101890

**Published:** 2020-10-15

**Authors:** Marta López-Alonso, Marta Miranda

**Affiliations:** 1 Department of Animal Pathology, Faculty of Veterinary Medicine, Universidade de Santiago de Compostela, Campus Terra, 27002 Lugo, Spain; marta.lopez.alonso@usc.es; 2 Department of Anatomy, Animal Production and Clinical Veterinary Sciences, Faculty of Veterinary Medicine, Universidade de Santiago de Compostela, Campus Terra, 27002 Lugo, Spain

**Keywords:** copper, cattle, supplementation, toxicity, deficiency

## Abstract

**Simple Summary:**

Copper supplementation in ruminants deserves special attention because of the narrow margin between deficiency and toxicity, both of which ruminants are susceptible to suffering from. Supplementation of copper above requirements to prevent deficiency has led to an increased number of outbreaks of copper toxicity being reported in recent years, particularly in dairy cattle. In this paper we describe the key points of copper metabolism in cattle that should be taken into consideration to guarantee an adequate copper supply while preventing toxic effects.

**Abstract:**

Ensuring adequate copper supplementation in ruminants is a challenging task due to the complexity of copper metabolism in these animals. The three-way interaction between copper, molybdenum and sulphur (Cu-Mo-S) in the rumen makes ruminants, particularly cattle, very susceptible to suffering from secondary copper deficiency. Paradoxically, excessive copper storage in the liver to prevent deficiency becomes a hazard when ruminants are fed copper-supplemented diets even slightly above requirements. While cattle were traditionally thought to be relatively tolerant of copper accumulation, and reports of copper poisoning were until recently somewhat rare, in recent years an increased number of episodes/outbreaks of copper toxicity in cattle, particularly in dairy cattle, have been reported worldwide. The growing number of lethal cases reported seems to indicate that copper intoxication is spreading silently in dairy herds, urging the development of strategies to monitor herd copper status and improve farmers’ awareness of copper toxicity. In fact, monitoring studies carried out on numerous samples collected from culled animals in slaughterhouses and/or diagnostic laboratories have demonstrated that large numbers of animals have hepatic copper concentrations well above adequate levels in many different countries. These trends are undoubtedly due to copper supplementation aimed at preventing copper deficiency, as dietary copper intake from pasture alone is unlikely to cause such high levels of accumulation in liver tissue. The reasons behind the copper overfeeding in cattle are related both to a poor understanding of copper metabolism and the theory of “if adding a little produces a response, then adding a lot will produce a better response”. Contrary to most trace elements, copper in ruminants has narrow margins of safety, which must also be formulated considering the concentrations of copper antagonists in the diet. This review paper aims to provide nutritionists/veterinary practitioners with the key points about copper metabolism in cattle to guarantee an adequate copper supply while preventing excessive hepatic copper loading, which requires à la carte copper supplementation for each herd.

## 1. Introduction

Copper (Cu) is an essential element for life and is required as a co-factor in hundreds of enzymatic reactions involved in red blood cell production, energy manufacturing, hormone formation, collagen synthesis and protection against oxidative damage. On the other hand, copper can be extremely toxic when present in excess, and thus all living organisms have developed specialized homeostatic mechanisms to recruit, deliver and eliminate copper and to neutralize its toxic effects [1,2]. In fact, most animal species, including humans, have efficient mechanisms for regulating copper stores, and they are therefore generally protected from excess dietary copper levels [3]; furthermore, copper has even been included at very high concentrations (up ten times the physiological requirements) as a growth promoter in pig diets [4].

Unlike other animals, ruminants do not have efficient regulatory mechanisms for copper, and episodes of chronic copper toxicity, particularly in sheep, have been described worldwide [5]. Ruminants are probably more susceptible to copper toxicosis than other species as an adaptation to grazing copper-deficient pastures, which in addition may contain antagonistic minerals, namely sulphur (S), molybdenum (Mo) and iron (Fe) [6]. As ruminants have poor homeostatic control over copper absorption, they have developed mechanisms for storing excess copper in the liver by decreasing copper excretion in bile. However, when exposed to copper concentrations above physiological requirements, ruminants do not modulate copper excretion in the bile and excessive hepatic copper accumulation occurs [5,7]. While acute copper toxicosis can occur after administration of a large dose of copper (often parentally due to a dosing error), copper toxicosis is usually a chronic process that occurs when excessive copper is supplied in the diet and hepatic copper reserves are overwhelmed [6]. Diagnosis of chronic copper toxicity is relatively easy in sheep and is normally indicated by haemoglobinuria, jaundice, methaemoglobinaemia and/or evidence of an acute haemolytic crisis and confirmed by high liver, kidney and serum copper concentrations [8]. By contrast, clinical signs in cattle are less evident, often of short duration, non-specific and would not immediately suggest chronic copper toxicity to the farmer, attending veterinarian or pathologist [9]. 

Among ruminants, sheep are the most susceptible to chronic copper toxicity, and farmers/nutritionists are aware that over supplementation with copper must be avoided to prevent excessive hepatic copper accumulation. However, this can be challenging as under some circumstances dietary copper requirements in sheep may overlap with levels that are toxic under other circumstances. For example, when sulphur and molybdenum are present at quite high levels in the diet, the copper requirement in sheep is 10 mg Cu/kg of diet. However, if molybdenum concentrations in the diet are low, dietary supplementation at 10 mg Cu/kg can lead to toxicity in some breeds [5]. 

By contrast, cattle were traditionally thought to be more tolerant of copper accumulation and reports of copper poisoning were, until recently, somewhat rare (possibly underdiagnosed [9]). However, in recent years, an increased number of episodes/outbreaks of copper toxicity in cattle have been reported worldwide, particularly in dairy cattle [9,10,11,12]. The growing number of lethal cases reported seems to indicate that intoxication is spreading silently in dairy herds, urging the development of strategies of monitoring herd copper status and amplifying the awareness of farmers about copper toxicity. In fact, monitoring studies carried out on numerous samples from culled cattle collected in slaughterhouses and/or diagnostic laboratories have demonstrated that a large number of cows have hepatic copper concentrations well above the adequate/normal/physiological levels, and that they are at risk of chronic copper toxicity in many countries such as the UK [9,11], New Zealand [12], the USA [13,14,15], the Netherlands [16] and Spain [17,18,19,20]. Dairy cattle are most affected, particularly Holstein Friesian [11,15] and Jersey cows [12,21], and available temporal data show an alarming tendency for an increasing incidence of cases [12]. These trends are undoubtedly due to copper supplementation aimed at preventing copper deficiency, as dietary copper intake from pasture alone is unlikely to cause such accumulation in liver tissue [22]. Recent research also indicates that copper already accumulates in bovine liver at dietary levels recommended by the industry, confirming that cattle are definitely less tolerant to copper than previously thought [23].

The reasons for copper over-supplementation in cattle are probably related to a poor understanding of copper metabolism in practice [24], together with the thinking along the lines of “if I add a little and I get a response then I will add a lot and get a better response” [11]. While most trace elements have large safety margins when supplemented in livestock [25,26], this is not true for copper in ruminants, which in addition must be formulated by taking into consideration the concentrations of antagonists in the diet. 

This review paper aims to provide nutritionists/veterinary practitioners with the key points about copper metabolism in cattle that should be taken into consideration to guarantee an adequate copper supply while preventing excessive hepatic copper loading. To achieve this, à la carte copper supplementation is required for each herd.

## 2. Copper Metabolism—Very Well Regulated in Most Animal Species

**Figure 1:**
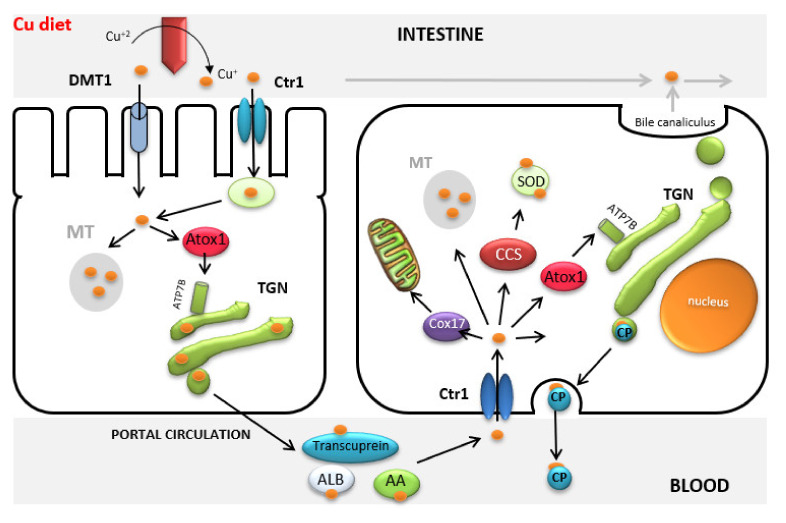
Copper transport. Ctr1: Copper specific transporter; DMT1: Divalent Metal Transporter 1; MT: Metallothionein; TGN: Trans Golgi Network; Cox17 and Atox1: Copper chaperone proteins; SOD: Superoxide dismutase; CP: Ceruloplasmin; CCS: Cytochrome c oxidase; ATP7B: Copper-transporting P-type ATPase.

At the cellular level, basic copper metabolism appears to be consistent throughout eukaryotes and can be traced from laboratory animals to humans through their shared evolution [27]. A large body of research has been conducted regarding cellular copper transport and liver metabolism, and numerous copper trafficking proteins have been identified (for a review, see [24,28,29,30]). Briefly, the first stage of copper uptake in the gut consists of the reduction from cupric to cuprous copper for translocation into the enterocyte (Figure 1). Most of the copper (ca.70%) at the brush border in intestinal cells is taken up by a specific copper transporter (Ctr1); the rest of the copper is taken up by the non-specific transporter Divalent Metal Transporter 1 (DMT1) and competes with other trace elements (iron and zinc). In the enterocyte, the copper chaperone proteins bind to and transport copper to other specific proteins or incorporate it into enzymes. When copper is present at concentrations above requirements, it enters the secretory pathway to bind to metallothionein in the Golgi body and is stored in the lysosomes, which thus protect the cell from free copper. Once metallothionein reaches saturation levels, copper continues through the secretory pathway from the Golgi body and exits the cells.

After efflux from the enterocytes, copper is bound to transcuprein (a specific copper carrier in plasma) and albumin to be transported from the gut through the systemic circulation to the liver. The liver plays an essential role in copper metabolism, in which a complex homeostatic control regulates copper secretion in the bile. Once copper enters the hepatocyte, it is reduced and transported in the cell by the same copper transporter (Ctr1) as in the enterocyte. Cytochrome c oxidase (CCS) and copper chaperone protein (Cox 17) then transport copper to the cytosol and mitochondrion respectively, whereas Atx1 transports copper to the Golgi body via ATP7B. ATP7B directs most of the copper to be incorporated into the ceruloplasmin before being returned to the circulation for distribution to other tissues. Ceruloplasmin is the predominant copper transporter in the systemic blood and after being synthesized in the liver is responsible for distribution of copper to the tissues. However, when copper-ceruloplasmin returns to the liver, the whole molecule is metabolized and is excreted through the bile. Finally, as in the gut, copper in excess to requirements is bound to metallothionein and enters the secretory pathway from the Golgi body and is stored in the lysosome, thus protecting the cell against free cellular copper.

## 3. Why is Copper Metabolism Different in Ruminants? The Difference Starts in the Rumen

Ruminants have a unique digestive system, which differentiates them from other mammals and which also affects copper metabolism. In ruminants, copper metabolism occurs within the rumen and is possibly the most spectacular example of how a nutritional interaction, i.e., the three-way interaction between copper, molybdenum and sulphur (Cu-Mo-S), can affect health [6,31]. This non-competitive interaction leads to a much lower level of intestinal absorption of copper (ranging from <1–10%) than in non-ruminant species and pre-ruminant calves (up to 70% [32,33]), but which varies greatly depending on the relative presence of the copper antagonists. When the diet contains high concentrations of copper antagonists, copper availability is very low. Despite adequate concentrations of copper in the feed, with estimated total copper background concentration in complete cattle feed (not mineral supplemented) of 6–11 mg/kg dry matter (DM) (for detailed information, as well as a review of individual data on feed materials, see European Food Safety Authority document [4]), secondary copper deficiency can occur [6], often justifying copper supplementation. However, in practice this “extra” copper supplementation related to the copper antagonists is very often unfounded, and in the absence of copper antagonists in the diet, chronic copper toxicity can occur.

**Figure 2:**
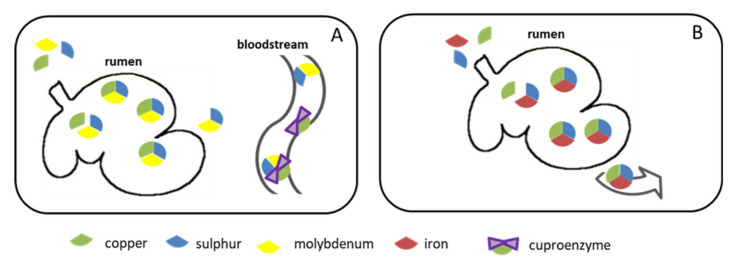
Interactions between copper and its antagonists. (**A**) In the rumen, molybdenum and sulphur combine to form thiomolybdates (MoS4), which display a high affinity for copper and will bind any copper present. If there is not enough copper in the rumen, MoS4 moves into the bloodstream and binds to cuproenzymes thus blocking them. (**B**) Iron and sulphur can also combine with copper in the rumen to create a stable, non-absorbable compound.

Inorganic and organic sulphur compounds are metabolized by microbes in the rumen, thus producing sulphide. Furthermore, sulphur and molybdenum react to form thiomolybdates (mono-, di-, tri- and tetrathiomolybdates). These compounds bind strongly to copper (tri- and tetrathiomolybdates bind copper irreversibly) to form copper thiomolybdates. The bound copper is insoluble, and is therefore not absorbed in the intestine. If there is no copper available in the rumen, the thiomolybdates will either be quickly absorbed through the rumen wall or will be absorbed more slowly via the small intestine and after that pass to bloodstream and can bind to copper in biological compounds [34] (Figure 2). 

The Cu:Mo ratio (mg/kg DM) can be used to predict the copper deficiency risk. In general, ratios <1 indicate a high risk of copper deficiency and ratios >3 are considered safe, although interpretation of the values can be affected by various factors. True copper absorption is expected to decrease by about 1% when the molybdenum concentration increases from 1 mg/kg DM to 5 mg/kg DM [33]. A concentration of molybdenum in the diet of ruminants of <1.5 mg/kg DM is generally observed; however, in some feed materials the content is relatively high (alfalfa 1.4–2.2; soybean meal 3–4; peas 3 mg Mo/kg DM) [35] and, for example, in grass the content varies from 0.9 to 5.4 mg Mo/kg DM, depending on the type of soil [33]. 

In addition to its role in the Mo-Cu interaction, sulphur can reduce copper bioavailability via formation of insoluble copper sulphide (CuS, Cu2S). A maximum tolerable level (MTL) of 4 g S/kg DM has been indicated for steers [36], although depression of copper absorption starts at 1 g S/kg DM [6]. Moreover, in steers, hepatic copper concentrations decreases from 230 to 140 or 96 mg Cu/kg DM as the concentration of sulphur increases from 0.12 to 0.31 or 0.46% DM [37]; similarly, in steers fed a diet containing 0.68% S in comparison with 0.24% S, plasma copper decreased from 16.7 to 11.8 µmol/l (1.07 to 0.76 mg/l) [38]. The amount of sulphur available to interact with copper is affected by different factors: Microbial degradation of sulphur compounds in the rumen, the levels of rumen degradable protein and fermentable carbohydrates and also the frequency of feeding, which affects ruminal pH (sulphide production increases when the rumen pH drops suddenly). Sulphur concentrations usually vary from 0.5–2 g/kg DM in feed. However, the values can be higher in, e.g., grass (1.8–4.3 190 g/kg DM [33]) and ethanol by-products (3–10 g/kg DM [38]). Water also contains variable amounts of sulphur, and sulphur intake from drinking water can reach 8–13 g/day when reference values for sulphate and sulphide are not exceeded [33]. Moreover, the effect of sulphur and molybdenum on copper availability varies depending on the feedstuff acting as a source of copper. Underwood and Suttle [39] reported that the amount of absorbable copper in ensiled grass was not greatly impaired by an increase in dietary molybdenum, but was greatly depressed by the addition of sulphur to the ration. When the diet included 0.2% sulphur, about 5.5% of the copper was available, but when the diet included 0.4% sulphur, the absorbable copper was reduced to about 1.5%. In hay, the inhibitory effect of molybdenum is present but is relatively small. As the sulphur content increases from 0.2 to 0.4% in hay, the absorbable copper decreases by 20–30%. The proportion of absorbable copper in fresh grass is lower than that of hay or ensiled grass at any given concentration of sulphur or molybdenum, and the addition of sulphur or molybdenum drastically decreases copper absorbability. Similarly, both sulphur and molybdenum also greatly affect copper absorption in concentrate-type diets. 

Copper absorption can be negatively affected by iron, which also participates in Cu-S-Mo interactions. High dietary levels of iron and sulphur enhance interactions between iron and copper. Iron can react with sulphur and copper in the rumen to generate iron sulphide or to produce the Fe-Cu-S complex, which decreases the availability of copper in the rumen for reacting with thiomolybdates. In the abomasum, sulphur from FeS reacts with copper to form insoluble CuS. The effects of iron on copper absorption do not therefore always involve molybdenum. Moreover, copper can also be absorbed by ferric oxide, thus reducing their absorption [33,34]. The effect of iron on copper absorption can be estimated from the Fe:Cu ratio, with values >100 indicating a high risk of copper deficiency and values <50 considered safe. In cattle, dietary concentrations of iron vary from 200 to 400 mg Fe/kg DM; however, the amount of iron varies greatly in the different components of feed (alfalfa 212–553; grass 149–443; wheat 57; rapeseed 82; rapeseed meal 499–534; mineral feed-phosphates 7000–15000 mg Fe/kg DM) [35] and, e.g., in grass, the iron content can be as much as 110-1400 mg Fe/kg DM depending on the type of soil [33]. Special care should be taken when crops are ensilaged as the acidification process may greatly increase iron bioavailability [40]. The overall dietary concentrations of iron, which will include dust or rain-splash soil, can range between 50 and 4000 mg Fe/kg DM [33]. 

Marginal ranges for copper antagonist concentrations in ruminant diets have been proposed to facilitate diagnosis of copper responsive disorders (CRD) [6] (Table 1).

## 4. Hepatic Copper Accumulation in Ruminants

**Figure 3:**
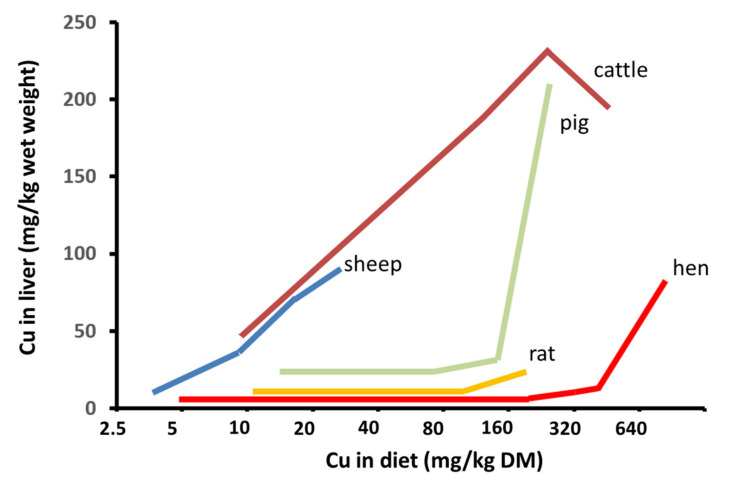
Species differences in responses in liver copper deposition to increased dietary copper supply. The figure accurately shows end points for liver copper in separate experiments in which dietary copper was varied: those end points reflect how long each study ran (adapted from Suttle [6] as suggested by the author, personal communication).

There are also major differences in copper metabolism in ruminants and non-ruminants in relation to the subcellular copper distribution within the hepatic cell and the capacity to excrete copper in the bile [41,42,43]. These differences form the basis of the level of tolerance of ruminants to copper toxicity, and they are thus used to establish the maximum tolerance levels in feed [5]. Biliary excretion of copper is adequate in non-ruminant species, such as pig and poultry, and most of the copper is bound to metallothioneins in the liver; these species can therefore tolerate high levels of dietary copper, and copper only begins to accumulate in the live after intake of high amounts of copper (more than 50 times the requirements) (Figure 3). Copper can be supplemented at very high concentrations in these species, e.g., to promote growth [4]. However, the capacity for copper biliary excretion is very limited in ruminants, and only a low proportion of copper is bound to metallothionein in the liver. This is observed in sheep, as dietary copper intake higher than requirements does not appear to increase biliary copper excretion [41,44], leading to deposition of very high concentrations of copper in the liver. Once this storage capacity is overloaded, a sudden and generally fatal haemolytic crisis occurs.

Because of the susceptibility of sheep to chronic copper toxicity and the clinical similarity to copper disorders in humans, hepatic copper metabolism has been widely studied in sheep [43,45,46,47,48,49] and susceptibility is considered to be related to the inability of sheep to accumulate large amounts of copper as metallothionein in the liver. While the role of metallothionein in copper metabolism has not been completely elucidated, it is generally considered to act as a storage buffer protecting the cell against free copper [24]. Studies of hepatic subcellular distribution in sheep [44,45,46,49] have shown that, as in most mammals, copper mainly accumulates in the cytosol bound to metallothionein during the early stages of copper accumulation; however, unlike other species, sheep have a limited capacity to accumulate large amounts of Cu-metallothionein in the liver, and saturation occurs very quickly. If there is a large influx of copper into the liver, the capacity of the metallothionein to bind copper and of the lysosomes to remove copper from the cytosol can be exceeded, and copper starts to accumulate at a higher rate in other organelles (mainly in the nucleus), while if accumulation is greater, copper may even remain as free copper ions in the cytosol; in both cases copper is responsible for major changes in liver structure and function [41,50,51].

Sequestration of excess copper by the proliferating lysosomes may maintain a constant concentration of copper in the cytosol of the liver cells [49]. At the beginning of this storage process, lysosomes increase greatly in number; however, as copper loading increases, lysosomal production may be significantly reduced (or may cease at a critical copper level) and excess copper may be accumulated in already present lysosomes, resulting in an increase in their volume [52]. The existing lysosomes may then become saturated and the concentration of copper in the cytosol and nucleus of these cells can, therefore, no longer be retained in a constant proportion and may rapidly rise to toxic levels. Although the mechanisms of liver necrosis in copper-loaded animals are not completely understood, it has been suggested that excess copper accumulation in the lysosome leads to rupture of the membrane, resulting in leakage of acid hydrolases into the cytoplasm and destruction of the liver cells [47]. However, it is also possible that accumulation of copper in the nuclear fraction destabilizes DNA and inhibits RNA polymerase activity, leading to nuclear disorganization and the subsequent death of the cells [41]. The increased concentrations of copper-free ions in the cytosol can also affect the metabolic activity in microsomes, cause lipid peroxidation of membranes and lead to degeneration and necrosis of the cells [49,53,54,55]. 

Information about copper toxicity in cattle is much more limited. However, earlier studies by our research group [56] indicate that cattle have a poor capacity to induce metallothionein and accumulate metallothionein-bound copper. In addition, copper distribution in the different subcellular fractions in cattle [57,58] is very similar to that described in sheep, in which the proportion of copper is generally highest in the large-granule fraction, followed by the cytosol and the nucleus, with only a small proportion in the microsomal fraction [44,45,46,49]. In many other mammalian species, most copper in the liver (i.e., 50% or more) occurs in the cytosol, and only a small proportion (20%) occurs in the large-granule fraction [44,45]. Differences in the intracellular distribution of copper in sheep, cattle and other species that are less susceptible to copper toxicity may be explained by the limited metallothionein-synthesizing capacity of ruminants [44]. 

In both cattle and sheep, the copper-accumulating capacity of the various subcellular fractions depends on the total liver copper concentration. The greatest difference is observed in the large-granule fraction, in which there is a gradual decrease in the rate of increase in copper concentration per unit increase in total liver copper concentration; the amount plateaus at around 450 mg/kg, i.e., at 4.5 times the generally accepted safe-adequate total liver concentration of copper in cattle [57]. Subcellular studies have shown that in sheep the large-granule fraction begins to plateau at hepatic copper levels of between 160 and 180 mg/kg wet weight, i.e., less than two times the safe-adequate concentration [44,45,46]. Other species such as rats are not as susceptible to copper toxicity, and the large-granule fraction becomes saturated at hepatic copper concentrations between 210 and 300 mg/kg wet weight, i.e., at almost 100 times the normal hepatic concentration [45,59]. This indicates that cattle may have a limited capacity to accumulate copper in the large-granule (i.e., lysosomal) fraction, although to a lesser extent than in sheep, and thus that saturation of the lysosomal copper compartment tends to occur at a lower total copper concentration than in other animals. 

**Figure 4:**
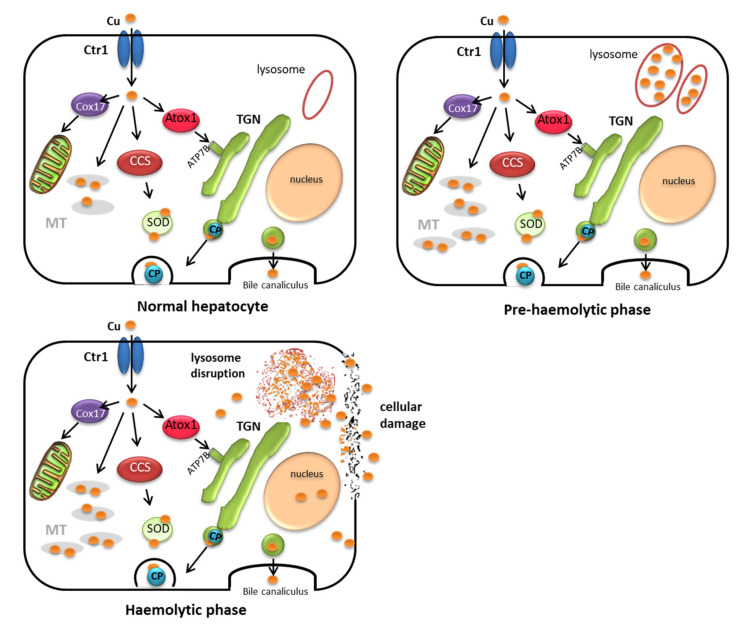
Phases of copper accumulation in the hepatocyte. Ctr1: Copper specific transporter; DMT1: Divalent Metal Transporter 1; MT: Metallothionein; TGN: Trans Golgi Network; Cox17 and Atox1: Copper chaperone proteins; SOD: Superoxide dismutase; CP: Ceruloplasmin; CCS: Cytochrome c oxidase; ATP7B: Copper-transporting P-type ATPase.

Finally, from a practical point of view it is important to consider that chronic copper poisoning is a two-stage process [52]. The first stage is the pre-haemolytic phase, during which lysosomes are able to sequester copper, which accumulates in the liver over a period of weeks or months without any signs of liver damage. However, once the lysosomes are overloaded, usually following some type of stressful event, storage of excess copper in the nucleus and cytosol causes lesions in the liver cells. A haemolytic crisis often occurs as copper is released from the liver (haemolytic phase) (Figure 4). The disease is generally clinically silent and basically undetectable until the haemolytic crisis occurs; in fact, during the pre-haemolytic phase of chronic copper toxicity there are generally no measurable effects on milk production, fertility or susceptibility to infectious diseases [9]. Indeed, recent findings indicate that in herds suffering from copper toxicity, the number of cases of copper poisoning greatly outnumbers the clinical cases reported [11,12,60]. 

In sheep, the factors precipitating clinical chronic copper toxicity are unclear, but mostly include stress, acute infections and poor nutrition [9]. In cattle, although traditionally only young calves were considered sensitive to copper, and adult cattle were thought to be quite tolerant to copper [9], recent episodes of chronic copper toxicity have mostly been seen in dry dairy cattle, and changes in social groups and the weight loss associated with liver catabolism triggered by abrupt withdrawal of concentrates have been suggested to be the main stressors [9,10].

## 5. Other Underlying Reasons: Breed Susceptibility

In sheep, breed is known to have a strong influence on susceptibility to copper disorders, and certain breeds have been classified as tolerant or resistant in terms of copper toxicity [39]; as a result, it has been possible to improve the resistance of sheep to copper deficiency and excess by appropriate cross breeding and selection programmes [61,62]. 

While cattle have been less extensively studied than sheep, the results of previous experimental studies in cattle have likewise indicated breed-related differences in copper metabolism. For example, it has been reported that some beef breeds, such as Simmental and Charolais, may have higher copper requirements than other breeds such as Aberdeen Angus [39,63] and that Simmental have lower plasma copper concentrations and lower apparent copper absorption and retention than Aberdeen Angus when fed diets not supplemented with copper [63,64,65]. In addition, the Limousin breed accumulates more copper in the liver than Aberdeen Angus, Simmental, Charolais and five other breeds (Braunvieh, Gelbvieh, Hereford, Red Poll and Pinzgauer) [66]. Furthermore, Jersey cows on copper-supplemented diets have higher plasma copper and ceruloplasmin activity levels than Holstein–Friesian cows [67]. These genetic differences in copper metabolism may be related to the efficiency of dietary absorption [66,67], biliary excretion of endogenous copper [68] or even the amount of feed intake [67]. Our experience with steers indicates that the higher hepatic copper accumulation in Holstein Friesian cows (dairy-aptitude) than in Galician blonde cows (a local beef-aptitude breed with a high muscular mass) [17] may be related to a lower physiological copper requirement due to the lower muscular mass in the former [19], thus leading to an excessive hepatic copper accumulation if copper supplementation is above requirements [20].

In practice, most clinical episodes of chronic copper toxicity have affected dairy herds (e.g., [9,69,70]), and biomonitoring of copper concentrations in the liver under the suspicion of copper over-supplementation has shown that most of the herds that exceed the safe limits are also dairy herds [11,12]. However, these findings do not necessarily indicate that dairy-aptitude cattle are more susceptible to copper toxicity than beef-aptitude cattle, and higher copper supplementation in dairy herds may also be an important factor. 

Within dairy herds, differences in the incidence of clinically affected individuals [9,70] and in the copper concentrations in the liver between breeds within the same farm [71] have been observed, with Jersey cows being more sensitive to excessive hepatic copper accumulation. Differences between both breeds probably occur at high dietary Cu supplementation, although the mechanism behind the higher hepatic copper accumulation in Jersey cows has not been elucidated [67].

## 6. Copper Requirements in Cattle and Copper Supplementation 

Accurate quantification of the trace mineral requirements of cattle is an extremely difficult task. Trace minerals are needed in minute amounts, but feed composition and dry matter intake can vary widely, making precise and accurate measurements of the intake of trace minerals difficult. For copper, the nutrient requirement model is generally used [72,73] in which the different factors (maintenance, lactation, reproduction and growth) are summed as needed. Afterwards, when evaluating or formulating a diet, the amount of mineral needed at the tissue level must be corrected by the corresponding absorption coefficient. 

As previously stated, the absorption coefficient of copper in ruminants varies greatly depending on the concentrations of the main copper antagonists molybdenum and sulphur, which must be taken into consideration when calculating the copper requirements. In general, National Research Council (NRC), as well as other nutritional scientific bodies, establish general copper requirements for different animal categories/ production levels (see Table 2) in diets where a low level of antagonism is expected (<1.5 mg Mo/kg DM; <0.2% S). However, when high concentrations of molybdenum and sulphur are present, the absorption coefficient should be corrected (NRC proposed using the equation developed by Suttle and McLauchlan [74]; for further details, see the review by NRC [72]). For example, increasing dietary sulphur from 0.2% (requirement) to 0.4% results in a 30 to 50% reduction in copper absorption when diets contain <2 mg Mo/kg DM. Thus, if a diet contains the equivalent of about 0.4% sulphur (including S from water), the dietary concentration of copper should be increased by 1.3 to 1.5 times. 

The absorption coefficient of copper could also be substantially reduced in pasture-fed animals as ingestion of soil reduces copper absorption [72]. If the entire diet comprises pasture, twice as much copper than recommended by the NRC might be needed. If pasture makes up 60% of the diet, an adjustment of 1.6 times might be considered. Cows can also ingest soil when eating silage or hay contaminated with soil. Moreover, other factors known to influence the absorption of copper is a high dietary intake of zinc, iron and calcium [72] and in certain extreme situations this should be also considered.

While all the above-mentioned factors influence copper availability, and equations have been derived to quantify their effects, in practice application of the models has not been always very successful. Overall, copper requirements in cattle will range in practical conditions from 5 to 20 mg/kg DM. However, in the total mixed ration (TMR) [21] and concentrate based diets [18,54], copper concentrations no higher than 5 mg/kg DM have been demonstrated to be sufficient to maintain an adequate copper status, while in situations with a large influence of copper antagonists copper concentrations up to 20 mg/kg DM are needed to prevent copper deficiency. The range may also be higher in situations of extreme exposure to copper antagonists, and the EU legislation allows for copper supplementation up to 30 mg/kg feed (88% DM), which equates to 40 mg/kg of the total dietary DM. 

## 7. What Happens in Practice? Are These Levels of Requirements Respected?

In practice, the mineral intake (including copper and its antagonists) is poorly documented in ruminant diets. The amount of trace elements present in feed ingredients of plant and animal origin is generally not taken into consideration in feed formulations, due to the enhanced costs of analysis. Moreover, no comprehensive data are available regarding their absorption from feed materials in ruminants. Therefore, there is a tendency to cover the physiological requirements by addition of mineral supplements to the ration. This is possible as trace elements are quite cheap and safety margins are generally wide. However, this is not the case with copper in ruminants, in which copper can readily reach toxic levels. 

The results obtained in field studies that measured copper concentrations in the diets of dairy herds in areas where over-supplementation of copper was suspected on the basis of high copper residues in the liver confirmed that copper over-supplementation is not justified by a high level of copper antagonists. For example, in a study conducted to determine the intake of minerals in commercial dairy herds in England [75], copper concentrations in excess of requirements were observed in early lactation, with a mean dietary copper concentration of 28 mg/kg DM (standard deviation 9.85), which is approximately 18 mg/kg DM above UK requirements, with 32 of the 50 herds feeding above the UK industry recommended maximum of 20 mg/kg DM. Although dietary mineral concentrations were generally lower in late lactation but still higher than requirements, two herds were fed above the legal limit and 27 herds above the UK industry guideline. The ratio between dietary molybdenum and copper concentration was low, and dietary molybdenum concentrations did not justify the high levels of copper being administered. In another study conducted in lactating dairy cows in California [76], it was found that the median copper concentration in the diet was about 1.9 times higher than the NRC requirement (9.5 mg/kg DM) but in 10% of the herds, the copper concentration was three times higher than the requirement. No correlations were found between dietary copper concentrations and dietary or total concentrations of the copper antagonists sulphur, molybdenum and iron that would justify over-supplementation of copper. In data collected across the EU in order to review the maximum authorized copper concentrations in feed [4], it was observed that 6.5% of the dairy cow rations exceeded the maximum current limit at that time (35 mg/kg DM); by contrast, none of the diets for fattening cattle exceeded this limit. 

## 8. How Can It be Established that A Herd is Being Over-Supplemented with Copper?

During the haemolytic stage of the disease, the most common indicators of chronic copper toxicity are increased levels of bilirubin in serum, the presence of haemoglobin in urine and methaemoglobinaemia. During the pre-haemolytic stage, ante-mortem plasma copper concentrations are slightly elevated, and increased serum concentrations of liver enzymes is one of the earliest biochemical changes observed. Once the haemolytic crisis is imminent, plasma copper concentrations reach maximum levels. During the clinical phase of copper poisoning, plasma or serum copper concentrations are usually higher than 3 mg/l [6]. An increase in serum concentrations of liver enzymes is one of the earliest biochemical changes in the pre-haemolytic phase, including elevated glutamate dehydrogenase (GLDH) aspartate aminotransferase (AST) and gamma-glutamyl transferase (GGT) concentrations [8]. Diagnosis of chronic copper toxicity is only confirmed by determining copper concentrations in both the liver and kidney. It is generally accepted that copper concentrations above 150 mg/kg wet weight in the liver (safe-adequate range: 25–100 mg/kg wet weight [77]) and 15 mg/kg wet weight in the kidney (reference range: 4–6 mg/kg wet weight) are indicative of toxicity [78]. 

However, identification of copper over-supplementation in cows during the silent chronic phase of hepatic copper accumulation is not an easy task as in the first stage there is no change in blood copper parameters. Serum/plasma copper concentration is a poor indicator of copper loading of the liver [79,80,81,82]; in fact, cows considered to be deficient or in the low-normal part of the reference range on the basis of evaluation of serum copper concentrations have been found to be at or near a toxic state on the basis of necropsy results or examination of hepatic biopsy specimens [83]. Acute hepatic necrosis only occurs when liver stores reach a critical threshold and liver copper stores are released causing transient high serum copper concentrations [84]. The whole-blood copper concentration (based on the ability of copper to bind with high affinity to some erythrocyte proteins [85] and the non-ceruloplasmin (CP) fraction [86,87] have been used as markers of copper in humans receiving high dietary copper as well as in patients with high copper loads (such as Wilson’s disease and other forms of childhood cirrhosis); however, neither of these have been demonstrated to provide an accurate prediction of hepatic copper accumulation in cattle [88]. Finally, although some authors have postulated that hepatic enzymes may be useful early markers during the long-term subclinical phase of hepatic copper accumulation [60,89], based on the fact that some cells undergo necrosis during this silent phase, leading to increased enzyme activity in the blood, other authors [55,88,90,91] have observed only weak correlations between serum enzyme activity and hepatic copper levels. During the chronic phase of progressive hepatic copper accumulation, only a few hepatocytes undergo necrosis at any specific time (0.45% of liver volume [47]), so that such elevations are transitory, and unless samples are obtained at least once a week, they may not be detected [52,92]. 

Liver biopsy seems to be the only diagnostic parameter able to identify those animals at risk of suffering from chronic copper toxicity. In practice, this can be done by percutaneous sampling, which has been demonstrated to be an affordable procedure at farm level [93]. Alternatively, post-mortem liver samples can easily be taken from culled cattle in abattoirs. This procedure allows large samples to be obtained, enabling histological study, and routine post-mortem testing also allows parallel evaluation of potential health risks to liver consumers [88]. In fact, post-mortem liver sampling in the slaughterhouse is recommended by EFSA for monitoring excessive copper accumulation in cattle [4]. Livers can be collected and frozen before analysis, enabling routine, affordable monitoring; multielement ICP techniques are nowadays routine in most analytical laboratories and enable the confirmation of copper loading, in addition to determining the levels of the copper antagonist in a single analysis. When considering histological study, copper loading can be demonstrated by using the rhodamine staining technique, which specifically stains copper-filled lysosomes. Qualitative rhodamine scoring mechanisms have been developed for dogs because canine liver biopsies are commonly evaluated histologically for liver pathologies [94]. It is worth noting that although a pattern of hepatic copper accumulation in copper-loaded cattle has been described (with the highest copper concentrations being found in the left lobe, followed by the processus papillaris, and the lowest copper concentrations found in the caudate and quadrate lobes) the samples obtained in vivo by percutaneous needle biopsy provided accurate estimates of overall hepatic copper status [95]. Finally, as there is wide variation in the hepatic copper concentrations within herds (up to 50% coefficient of variation according to Grace et al., [96]), a sufficient number of cows should be sampled per herd. 

## 9. What Is the Limit Between Safe Copper Storage and Hazard Overloading?

Traditionally there seemed to be consensus in the scientific literature as regards establishing the upper range of adequate hepatic copper concentrations in cattle at around 100 mg/kg fresh weight (adequate range: 25–100 mg/kg wet weight according to Puls [77]; 6–95 mg/kg according to Suttle [6]) and up to 140–150 mg/kg, the concentration at which cattle would be at risk of suffering from copper toxicity [6,8,77]. This small margin of safety was not considered of concern as cattle were thought to be relatively tolerant to copper toxicity and that episodes of copper toxicity were quite rare. However, opinion has changed since more episodes of chronic copper toxicity have appeared in dairy herds.

Recent studies indicate a large variability in the hepatic copper concentrations associated with toxicity in cattle, as well as the levels of copper accumulation in individuals within the same herd. Case studies of herds suffering from chronic copper toxicity have reported variable hepatic copper concentrations in cattle succumbing to chronic copper toxicity and those not exhibiting any clinical signs. Bidewell et al. [9] recorded an episode of chronic copper toxicity in which the affected cows had hepatic copper concentrations ranging from 145 to 418 mg/kg wet weight (mean 253 mg/kg), whereas the non-clinically affected cows had hepatic copper concentrations within this range (up to 389 mg/kg). Similarly, in another episode of chronic copper toxicity in Jersey cattle in New Zealand, post mortem analysis revealed hepatic concentrations (ranging between 146 and 172 mg/kg wet weight) within the same range as the non-clinically affected animals in the same herd (ranging from 121 to 222, mean value 166 mg/kg wet weight) [70]. These studies illustrate that a high liver copper concentration per se does not necessarily cause chronic copper toxicity, but represents a potential hazard that can be manifested atypically in casualties (young or old) by exposure to stressors such as abrupt dietary change or acute infections [97]. The risk of excessive hepatic accumulation may be at least partly related to the way in which copper is accumulated within the different compartments of the hepatic cells. Thus, once the capacity of the lysosomes to accumulate copper reaches a plateau, the animals are at risk of suffering a haemolytic crisis, and the capacity of the animal to co-neutralize copper bound to metallothionein decreases, as already stated. These factors may vary significantly within individuals and possibly within different breeds, as well as in relation to other dietary components. For example, as metallothionein concentrations within the hepatic cell are closely related to the zinc status of the animal, zinc supplementation could protect individuals against chronic copper toxicity [98].

This new scenario calls into question the practice of feeding cattle (or sheep) in a way that leads to accumulation of such high concentrations of copper in their livers. As far we are aware, apart from ruminants, no other mammals, including humans, accumulate such high concentrations of copper in the liver (normal hepatic concentrations are at least one order of magnitude lower [77]). It is only in cases involving genetic disorders—the most common being Wilson’s Disease in humans [99] and copper-related hepatitis in some breeds of dogs [100,101]—when excessive hepatic copper accumulation leads to severe liver damage, similar to that observed in copper-loaded sheep and cattle.

Cattle should be given copper supplementation (if needed) only to cover the physiological requirements and guarantee adequate productive (milk of beef) and reproductive performance. According to Grace and Knowles [12] under farming conditions in New Zealand, supplementing dairy cows with copper when liver concentrations are higher than 6 mg/kg wet weight is unlikely to benefit performance, and relatively low levels of copper are required to achieve this. Whether the findings of the aforementioned researchers for the New Zealand dairy herd apply to more intensive farm production is not known, because studies evaluating copper dose-response and animal performance are very scarce. Most studies have shown that copper inputs positively affect hepatic copper balance in cattle, but liver loading by itself does not have intrinsic physiological benefit, economic value or equate to farm profitability. In our opinion, copper supplementation studies on herds of differing hepatic copper status are needed, using well-characterized low-copper diets and assessing the impact of supplementation on productive and reproductive performance. The findings of such studies could determine the minimum dietary copper requirements and may demonstrate that supplementation producing high hepatic copper loading does not actually improve performance. 

## 10. What Should I Do Once I Know When My Cattle are Accumulating Copper in the Liver?

The first thing to do when chronic copper toxicity (or excessive hepatic copper accumulation) is diagnosed in a herd is to identify and withdraw the source of copper. As main feed ingredients/pasture do not usually contain excessive copper (background copper concentration of complete feed: 6-11 mg/kg DM [4]), the most likely reason will be copper over-supplementation within the mineral supplement. Some cases of copper poisoning have been caused by an error in the copper supplementation or simply by given ruminants concentrate feed destined for other animal species such as pigs [102,103,104]. On other occasions the reason may be an ingredient/supplement containing a highly available source of copper, e.g., the palm kernel cake given to dairy cattle as a protein supplement [10,69]. In some cases, this information would be sufficient to identify and redefine excessive copper supplementation. In order to fine-tune the quantity of copper needed to meet the requirements while preventing excess hepatic copper accumulation, a detailed analysis of the complete ration would be ideal, taking into consideration the concentrations of copper and its antagonists and also the bioavailability of copper from the feed materials, without forgetting drinking water, which may represent a significant source of iron and sulphur for cows [33]. If low levels of copper antagonists are identified and/or copper is rather available (i.e., TMR or highly concentrate feed [105]), it is possible that no, or less, copper supplementation will be needed. 

Once the source of copper has been withdrawn, treatment of copper-overloaded animals aims to either bind the copper within the body, particularly the liver, or to create a copper-deficient status, by increased utilization as well as hepatic excretion of copper [106]. Both aims can be achieved by treatment with molybdenum salts, which bind with copper to form insoluble complexes and either render systemic copper non-toxic or reduce the availability of enteric copper, and have thus been extensively used in chronic copper-poisoned sheep [6]. While parenteral/subcutaneous administration of tetrathiomolybdate salts is the best treatment for clinically affected animals, as these salts bind to systemic copper, oral treatment with molybdenum salts, such as sodium molybdate, is useful in animals with subclinical hepatopathy as it directly reduces the availability of enteric copper [97]. 

While more limited, there is also experience on the use of molybdenum salts to treat chronic copper toxicity in cattle. In an outbreak of copper poisoning in a herd of 250 Jersey cows in New Zealand [70], each the affected cows was supplemented with 0.5 g ammonium molybdate and 1 g sodium sulphate, via an oral drench, for five days, and the herd was given no supplementary feed or mineral supplements; the mean concentration of copper in liver 44 days after the start of treatment was ca. 112 mg/kg wet weight, which was on average 37% lower (range 15–54%) than at the start of the treatment. In response to a similar case in a herd of 350 Jersey cows, also in New Zealand, the herd was orally treated with 500 mg/cow/day of sodium molybdate for five weeks, together with the removal of all copper supplementation [69] resulting in a mean reduction of 57% (range 38–71%) of the hepatic copper concentrations 26 days after the start of the treatment. In Israel, an episode of chronic copper toxicosis occurred in a Holstein–Friesian dairy herd that was accidently supplemented with 250 mg copper /kg complete feed for 6 months, resulting in a mean liver copper concentrations of 405±120 mg/kg wet weight. Molybdenum treatment consisting of 200 mg Mo/cow/day for seven days, together with removal of all copper supplementation, resulted in complete recovery of the dairy herd, with milk production returning to the normal range within two to three months [103]. However, it is important to consider that none of these case reports included a control group. Thus, some, or most, of the observed reductions in concentrations of copper in liver may have been due to the cessation of copper supplementation rather than to the molybdenum treatment. In a study including control groups in which yearling Friesian bulls on two farms were suffering from chronic copper poisoning and were treated with sodium molybdate (3 mg/kg live weight on farm A and 7 mg/kg live weight on farm B) administered as a drench at weekly intervals for four weeks [106], the effect regarding the reduction in copper concentration was better in the control group on both farms (farm A: 55 vs. 27% (initial copper in the liver: 145 mg/kg wet weight); farm B: 25 vs. 15% (initial copper in the liver: 276 mg/kg wet weight) for control and treated respectively). These unexpected results were possibly related to an adequate dose/administration period: It is possible that excess thiomolybdate that did not chelate copper in the intestine was absorbed and bound to organic copper, thus preventing copper excretion by the bile. Moreover, when treating asymptomatic animals in a herd clinically affected by chronic copper poisoning, it is important to consider that hepatic copper concentrations usually vary widely in cattle. If all cows are given the same dose of sodium molybdate, extrahepatic side effects (e.g., repeated large parenteral doses of sodium molybdate have caused pituitary endocrinopathy in sheep [107]) may be most marked in the individuals at lowest risk. 

## 11. A Brief Note on Other Ruminant Species

Once more it has been demonstrated that goats are not sheep, with large differences being observed between these ruminant species in terms of copper requirements and copper tolerance. It has previously been established that copper requirements are higher in goats (15–25 mg/kg DM for kids and adults, respectively) than in sheep (5––8 mg/kg DM [108]). Some studies [109,110,111] suggest that copper requirements may be even higher in caprine species and, that under some circumstances, they may obtain benefits from copper supplementation above NRC requirements. This observation was reported by the EFSA in a revision of the currently authorised maximum copper content in complete feed [4] and has led to a proposed increase in the maximum allowed concentrations of copper to 35 mg/kg diet [112]. On the other hand, goats can tolerate much higher dietary copper concentrations than sheep. Long term experimental studies in which animals were fed diets containing copper concentrations up to 640 mg/kg DM [113] did not find any signs of copper toxicity in goats. This high tolerance to copper could be explained by a lower hepatic uptake than in sheep (six to nine times [114]). Moreover, goats can tolerate much higher levels of the copper antagonist molybdenum than cattle and sheep, without suffering secondary copper deficiency [115]. Both factors together make copper supplementation easier in goats.

Within wild ruminant species, deer deserve a special mention as these animals are increasingly being maintained in captivity for commercial (e.g., venison and antler production) and recreational purposes; consequently, safe and effective feeding practices are needed to ensure animal health and productivity [116]. Information on copper requirements in deer is very scarce. Using a factorial model, Grace et al. [117] estimated the dietary copper requirements for growing hinds to be 8 mg/kg DM. However, there may be some breed-related differences in copper requirements that justify further research; e.g., wapiti have higher copper requirements and display higher susceptibility to copper deficiency than red deer. Moreover, deer seem to be less susceptible to copper-molybdenum antagonism than cattle [118] and can tolerate copper concentration in the diet up to 200 mg/kg DM for long periods without showing signs of copper toxicity [119]. This information (although limited) indicates that copper supplementation in deer is less challenging than in cattle and sheep.

## 12. Conclusions

Adequate copper supplementation in ruminants is challenging because of the complexity of copper metabolism in these animals. The three-way interaction between copper, molybdenum and sulphur (Cu-Mo-S) within the rumen makes ruminants, particularly cattle, very susceptible to suffering from secondary copper deficiency. Ruminants have evolved a system for storing copper in the liver through a low capacity for biliary elimination. Paradoxically, storage of excessive copper in the liver becomes a high risk when ruminants are fed copper diets slightly above requirements. Under practical conditions, total copper concentrations in the diet associated with copper deficiency in the presence of high concentrations of copper antagonists can lead to chronic copper toxicity when copper availability is high. 

The narrow margin of safety between hepatic concentrations of copper considered adequate and toxic makes the design of an effective and customized copper supplementation strategy for cattle on different farms essential. Unlike most essential trace elements, the available information on copper concentrations in feedstuff is not sufficient to ensure adequate copper supply in cattle diets, and it is essential to consider the concentrations of main copper antagonists (molybdenum, sulphur and iron), the type of diet (pasture, silage, hay, concentrate TMR) and also the breed. However, this is not always easy in practice, and direct measurement of animal copper status to confirm the level of stored copper in the liver should be carried out before increasing/changing copper supplementation as well as to ensure adequate copper supplementation. 

Finally, scientists and veterinary practitioners should question the reportedly adequate copper reference ranges in the liver (up to 100 mg Cu/kg fresh weight). What concentration of copper in the liver no longer represents safe storage and constitutes a risk? We should challenge the existing copper reference ranges and dietary requirements, encourage monitoring of the copper status of herds and organize more dairy cow production-response trials under meaningful farming conditions. Such actions together with informed decisions will help to optimize copper supplementation in cattle and reduce the risk of chronic copper toxicity.

## Figures and Tables

**Table 1:** Marginal bands for copper antagonist concentrations in the diet to aid the diagnosis of copper response disorders (CRD) in cattle fed on fresh herbage or forage-based diets (adapted from Suttle [6]). Marginal bands indicate the probability of CRD, which increases with proximity to the value shown in bold.

Parameter	Diet Based on	Marginal Bands	Interpretive Limit
Cu/Mo	Herbage	**1.0**–3.0	Diet S ˃2 g/kg DM ^1^
	Forage	**0.5**–2.0	Diet Mo <15 mg/kg DM
Fe/Cu	Herbage	50–**100**	
Cu	Herbage	**6**–8	Diet Mo <1.5 mg/kg DM
	Forage	**4**–6	

^1^ DM: Dry matter

**Table 2:** Copper requirements (mg/kg DM complete feed) and maximum copper authorised in the European Union for bovines.

Organization	Calves Pre-Rumination	Beef	Dairy
**NRC ^1^**	10	10	10–18
**GfE ^2^**	−	8–10	10
**CVB ^3^**	10	11–13	7–17
**INRA ^4^**	−	10	10
**Maximum authorized EU ^5^**	15	30	30

^1^ NRC: National Research Council, USA [36,72]; ^2^ GfE: Gesellschaft für Ernährungsphysiologie, Germany [74,75]; ^3^ CVB: Centraal Veevoederbureau, the Netherlands [33]; ^4^ INRA: Institut National de la Recherche Agronomique, France, [76]; ^5^ EU: European Union.
